# CRISPR/nCas9-Edited CD34+ Cells Rescue Mucopolysaccharidosis IVA Fibroblasts Phenotype

**DOI:** 10.3390/ijms26094334

**Published:** 2025-05-02

**Authors:** Angélica María Herreno-Pachón, Andrés Felipe Leal, Shaukat Khan, Carlos Javier Alméciga-Díaz, Shunji Tomatsu

**Affiliations:** 1 Nemours Children’s Health, Wilmington, DE 19803, USA; angelicamaria.herrenopachon1@nemours.org (A.M.H.-P.); andres.lealbohorquez@nemours.org (A.F.L.); shaukat.khan@nemours.org (S.K.); 2 Faculty of Arts and Sciences, University of Delaware, Newark, DE 19716, USA; 3 Institute for the Study of Inborn Errors of Metabolism, Faculty of Science, Pontificia Universidad Javeriana, Bogotá 110231, DC, Colombia; cjalmeciga@javeriana.edu.co; 4 Department of Pediatrics, Graduate School of Medicine, Gifu University, Gifu 501-1193, Japan; 5 Department of Pediatrics, Thomas Jefferson University, Philadelphia, PA 19107, USA

**Keywords:** CD34+ cells, gene editing, CRISPR/nCas9, MPS IVA

## Abstract

Mucopolysaccharidosis (MPS) IVA is a bone-affecting lysosomal storage disease (LSD) caused by impaired degradation of the glycosaminoglycans (GAGs) keratan sulfate (KS) and chondroitin 6-sulfate (C6S) due to deficient N-acetylgalactosamine-6-sulfatase (GALNS) enzyme activity. Previously, we successfully developed and validated a CRISPR/nCas9-based gene therapy (GT) to insert an expression cassette at the AAVS1 and ROSA26 loci in human MPS IVA fibroblasts and MPS IVA mice, respectively. In this study, we have extended our approach to evaluate the effectiveness of our CRISPR/nCas9-based GT in editing human CD34+ cells to mediate cross-correction of MPS IVA fibroblasts. CD34+ cells were electroporated with the CRISPR/nCas9 system, targeting the AAVS1 locus. The nCas9-mediated on-target donor template insertion, and the stemness of the CRISPR/nCas-edited CD34+ cells was evaluated. Additionally, MPS IVA fibroblasts were co-cultured with CRISPR/nCas-edited CD34+ cells to assess cross-correction. CRISPR/nCas9-based gene editing did not affect the stemness of CD34+ cells but did lead to supraphysiological levels of the GALNS enzyme. Upon co-culture, MPS IVA fibroblasts displayed a significant increase in the GALNS enzyme activity along with lysosomal mass reduction, pro-oxidant profile amelioration, mitochondrial mass recovery, and pro-apoptotic and pro-inflammatory profile improvement. These results show the potential of our CRISPR/nCas9-based GT to edit CD34+ cells to mediate cross-correction.

## 1. Introduction

Mucopolysaccharidosis (MPS) IVA (OMIM: 253000) is a genetic lysosomal storage disease (LSD) caused by a deficiency in the N-acetylgalactosamine-6-sulfatase (GALNS) enzyme that leads to the lysosomal accumulation of the glycosaminoglycans (GAGs) keratan sulfate (KS) and chondroitin 6-sulfate (C6S) [1,2,3,4,5,6]. The accumulation of KS and C6S disrupts the function of chondrocytes in the growth plates, impairs cartilage matrix development, and inhibits bone matrix mineralization. The resultant incomplete or defective endochondral ossification and successive imbalance of growth in MPS IVA patients cause unique skeletal symptoms such as prominent forehead, abnormal face with a large mandible, disproportionate short-trunk dwarfism with short neck, cervical spine instability with odontoid hypoplasia, cervical spinal cord compression, pectus carinatum, tracheal deviation and obstruction, restrictive lung, flaring of the rib cage, kyphoscoliosis, platyspondyly, hip dysplasia with coxa valga, genu valgum, hypermobile joints, waddling gait, and pes planus. The degree of imbalance of growth in bone and other organs and tissues largely contributes to unique skeletal dysplasia and clinical severity, including narrowing trachea [1]. Currently, an enzyme replacement therapy (ERT) is approved to treat MPS IVA patients [7]; nevertheless, ERT has several limitations: (i) it has a limited impact on skeletal dysplasia [8,9,10,11,12,13,14], (ii) infused enzymes are unstable and taken up mainly by the liver and spleen in rapid clearance [8,15], (iii) most patients with ERT produce antibodies against the enzyme, developing immune reactions [16,17,18,19,20,21], and (iv) ERT costs $500,000/year/25 kg in the USA [3,14,22,23]. Allogeneic hematopoietic stem cell (HSC) transplantation is a therapeutic option available for patients with MPS IVA [24,25,26,27,28,29,30]; nevertheless, the risk of development of graft versus host disease (GVHD) remains a significant concern with a limited bone impact and absent a human leukocyte antigens (HLA)-matched donor [25]. Other approaches, including pharmacological chaperones (PC) [31,32] and substrate degradation therapy (SDT) [33], have been tested and could turn into novel therapies shortly.

Classical [34,35] and CRISPR/Cas9-based [36,37,38] gene therapy (GT) alternatives have been tested using in vitro and in vivo models of MPS IVA with encouraging results. Regarding the CRISPR/Cas9 system, we reported the use of a novel strategy using Cas9 Nickase (nCas9) to introduce double nicking at the AAVS1 locus to mediate homologous recombination of an expression cassette carrying a wild-type (WT) cDNA of GALNS [37]. Our in vitro data showed a significant phenotype recovery in MPS IVA fibroblasts carrying different mutations in the GALNS gene [37,38]. Likewise, when evaluating the CRISPR/nCas9 system in MPS IVA mice, a significant improvement in the mono-sulfated KS levels was achieved upon direct infusion using iron oxide nanoparticles as non-viral vectors [36]. Despite these promising findings, direct injection of the CRISPR/nCas9 system partially rescued bone lesions [36], suggesting that novel bone-impact approaches must be explored.

In 2019, Gomez-Ospina et al. successfully implemented an ex vivo-based CRISPR/Cas9 GT in MPS I mice [39]. The CRISPR/Cas9 system was targeted to the human CCR5 locus in hematopoietic stem progenitor cells (HSPCs) to insert an expression cassette containing IDUA cDNA, which is impaired in MPS I. CRISPR/Cas9-edited HSPCs showed stable gene insertion while preserving stemness and engraftment potential [39,40]. Most interestingly, CRISPR/Cas9-edited HSPCs normalized femur, zygomatic, and skull bone thickness, suggesting that ex vivo CRISPR/Cas9-mediated GT could be a potential alternative in treating bone manifestations in MPS. Consequently, using the CRISPR/Cas9 system to insert expression cassettes in MPS IVA patient-derived HSPCs could represent a novel alternative used to overcome GVDH while inducing the expression of the functional GALNS enzyme, which can correct bone pathology and systemic manifestations of the disease.

Ex vivo lentiviral (LV) GT was recently tested in MPS IVA mice by Celik et al. and reported in 2024 [35]. Interestingly, while ex vivo LV GT resulted in higher GALNS expression in plasma and tissues [35] than observed in our studies using the CRISPR/nCas9 system [36], both LV- and CRISPR/nCas9-mediated GT partially recovered the bone pathology. Although it was not evaluated by Celik et al. (2024) [35], LV-based GT raises several concerns due to the LV random integration, which could increase the potential risk of oncogenesis [41,42]. Early ex vivo LV-mediated GT in two patients suffering from X-linked chronic granulomatosis resulted in myelodysplasia or acute myeloid leukemia due to insertional activation of oncogenes [43]. Clinical trials recently observed similar hematological findings (NCT01896102, NCT03852498, and NCT02698579) for cerebral adrenoleukodystrophy with ex vivo LV GT [44]. These unwanted effects can be significantly reduced using the CRISPR/Cas9 system due to the high precision during gene insertion [45,46,47,48] and the elimination of off-target effects when using nCas9 [37,49,50].

In light of this evidence, in this study, we first assessed the effectiveness of our CRISPR/nCas9 system in editing CD34+ hematopoietic stem cells (HSCs) and proving the cross-correction of MPS IVA fibroblasts.

## 2. Results

### 2.1. CRISPR/nCas9-Based Genome Editing System Efficiently Preserves the Self-Renewal and Differentiation Potential of CD34+ Cells

**Figure 1:**
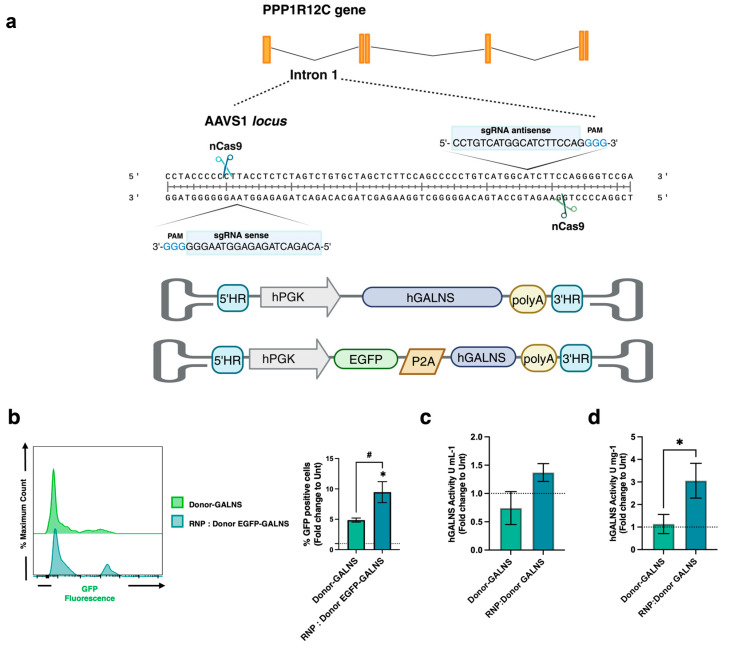
CRISPR/nCas9-based genome editing in CD34+ cells. (**a**) Schematic representation of CRISPR/nCas9 system strategy and two sgRNAs, targeting the AAVS1 locus and PAM sequence (blue) (top). AAV6-donor template construct. Two homologous arms flank an expression cassette containing an hPGK promoter, GALNS cDNA, and the bovine growth hormone (bGH) poly(A) signal sequence (bottom). Figure created with BioRender.com. (**b**) Representative histogram from CD34+ cells positive for GFP (left). The percentage of GFP-positive CD34+ cells with AAV6-donor-EGFP and with or without the RNP-based CRISPR/nCas9 or (right) (*n* = 6). (**c**) Extracellular and (**d**) intracellular GALNS activity in CD34+ cells with the RNP-based CRISPR/nCas9 or AAV6-donor (*n* = 5). Results are expressed as fold-change relative to untransfected CD34+ cells. The dotted line represents the untransfected CD34+ cell levels detected. * *p* < 0.05, ^#^ *p* < 0.001.

Previously, we reported the CRISPR/nCas9 system using two sgRNAs that target the AAVS1 locus to insert an expression cassette containing GALNS cDNA via non-viral vectors [37]. In this study, we evaluated an RNP-based CRISPR/nCas9 system to mediate the homologous recombination (HR) of the expression cassette using AAV6 as a viral vector (Figure 1a). Initially, HEK-293 cells were transfected with the RNP-based CRISPR/nCas9 system at various molar ratios (nCas9:sgRNA) over 6, 12, and 24 h to optimize nCas9 cutting. The nCas9-dependent DNA cutting was determined by a mismatch detection strategy using the T7 endonuclease assay. Under our experimental conditions, we found an on-target efficiency of 31.9% at a 1:2.5 molar ratio (Supplementary Figure S1a). Then, we tested the transduction efficiency of the AAV6-donor template at different MOIs. HEK-293 cells were transduced with AAV6-EGFP/GALNS, and the percentage of cells expressing the reporter GFP was analyzed by flow cytometry (Supplementary Figure S1b). An MOI of 15,000 exhibited the highest transduction rate, as observed via EGFP expression.

Following our optimization experiments, we edited CD34+ cells using the RNP complex and AVV6-donor-EGFP at a 1:2.5 molar ratio and 15,000 MOI, respectively, and analyzed them after 30 days of culturing. As shown in Figure 1b, AAV6-donor-EGFP transduction in the absence of the RNP complex resulted in a 5-fold increase in the EGFP expression compared to untreated CD34+ cells. When CD34+ cells were edited by electroporating the RNP complex followed by AAV6 transduction, we observed a 9-fold increase in EGFP-positive cells relative to untreated CD34+ cells 30 days post-editing, suggesting that the RNP complex led to a better expression of EGFP by the integration of our expression cassette in the CD34+ cells. Further assessing the therapeutic effectiveness of RNP-based CRISPR/nCas9 system and AAV6-donor transduction, we evaluated genome editing efficiency by measuring GALNS enzyme activity in CRISPR/nCas9-edited CD34+ cells (Figure 1c,d). While AAV6-donor transduction alone resulted in a 0.7-fold and 1.1-fold increases in the extra- and intra-cellular GALNS enzyme activity, respectively, the RNP complex electroporation followed by AAV6-donor transduction notably increased the extra- and intra-cellular GALNS enzyme activity, with up to 1.4- and 3.1-fold increases observed in CD34+ cells (Figure 1c,d).

**Figure 2:**
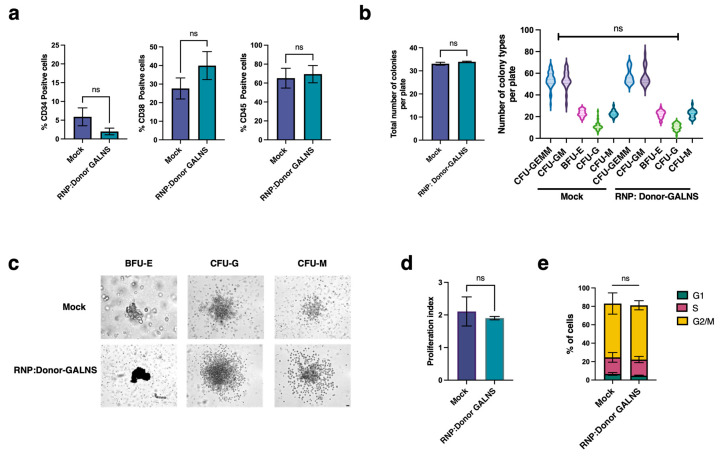
Stemness evaluation of CRISPR/nCas9-edited CD34+ cells. (**a**) Percentage of positive cells for CD34, CD38, and CD45 (*n* = 6). (**b**) Differentiation potential was detected by the expression level of the differentiation markers CD15, CD14, and CD235a (*n* = 12). Total number of colonies formed per plate (left) and the number of colony types of progenitor cells and mature multilineage colonies (right). (**c**) Phase-contrast microscopy of representative BFU-E, CFU-G, and CFU-M in CD34+ cells transfected with or without CRISPR/nCas9. Scale bar, 50 µm (**d**) Proliferation index (*n* = 5). (**e**) Cell cycle distribution (*n* = 5). Mock: Unedited CD34+ cells. ns: no significant differences.

To evaluate the impact of the RNP-based CRISPR/nCas9 system on the stemness of CD34+ cells, we assessed the stem properties (differentiation and self-renewal) of CRISPR/nCas9-edited CD34+ cells 14 days post-editing. First, we examined the frequency of CD markers and the expression profiles of primitive hematopoietic progenitor cells (CD34+, CD38+, and CD45+) by flow cytometry (Figure 2a). We found no significant changes in the frequencies of the CD34+, CD38+, and CD45+ markers in CRISPR/nCas9-edited CD34+ cells compared to untransfected cells 14 days post-editing (Figure 2a). Next, we evaluated the differentiation potential of CRISPR/nCas9-edited CD34+ cells by assessing colony-forming units (CFUs) through flow cytometry and microscopy assessment. We observed no difference in the number of colonies per plate or colony types between CRISPR/nCas9-edited CD34+ cells and untransfected cells 14 days post-editing using either method (Figure 2b,c). Through flow cytometry analysis using the differentiation markers CD15, CD14, and CD235a, we determined that CRISPR/nCas9-edited CD34+ cells retained the capacity to differentiate into multipotent progenitor cells, including the CFU-GEMM (multipotential colony-forming cells), and committed progenitor cells, such as CFU-GM (Colony Forming Unit-Granulocyte–Macrophage) and BFU-E (Erythroid Burst-Forming Units). Additionally, we detected differentiation into mature multilineage colonies, such as CFU-G (Granulocyte Progenitor Cells) and CFU-M (Monocytic Progenitor Cells) (Figure 2b), suggesting that our CRISPR/nCas9 system does not affect the stemness of CD34+ cells.

Using flow cytometry, we also assessed the proliferation ability and cell cycle preservation in CRISPR/nCas9-edited CD34+ cells. No significant differences were observed in the proliferation index (Figure 2d) or cell cycle frequency (Figure 2e) 14 days post-editing. Although a decrease in cell viability was observed on day 4 post-treatment, we found that CRISPR/nCas9-edited CD34+ cells exhibited a faster recovery over time, compared to CD34+ cells transduced with AAV6-donor alone (Supplementary Figure S2), highlighting that AAV6 alone can increase cell toxicity. These results demonstrate that our RNP-based CRISPR/nCas9 system successfully increases GALNS enzyme levels in CD34+ cells, preserving their stemness after long-term post-editing.

### 2.2. Therapeutic Efficacy of CRISPR/nCas9-Edited HSCs in MPS IVA Fibroblasts Co-Culture

**Figure 3:**
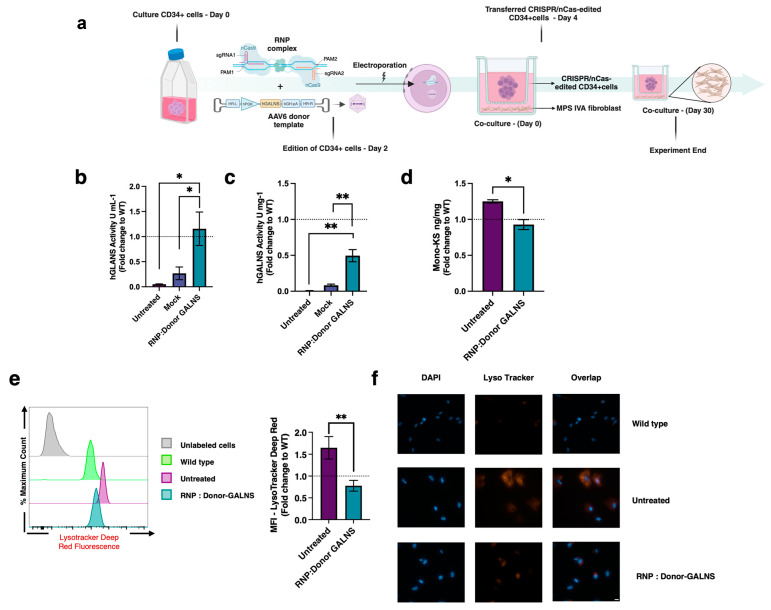
GALNS enzyme activity and lysosomal mass evaluation in MPS IVA fibroblasts co-cultured with CRISPR/nCas9-edited CD34+ cells. (**a**) Schematic representation of the experimental process. CD34+ cells were electroporated with a ribonucleoprotein (RNP) complex comprising CRISPR/nCas9 and two sgRNAs targeting the AAVS1 locus at a concentration of 1:2.5 molar ratio. We transduced the AAV6 donor template at an MOI of 15,000. After 48 days, MPS IVA fibroblasts were co-cultured with CRISPR/nCas9-edited CD34+ cells for up to a month. Figure created with BioRender.com. (**b**,**c**) Extracellular and intracellular GALNS activity in MPS IVA fibroblasts (*n* = 6). Mock: Unedited CD34+ cells. (**d**) Mono-sulfated KS levels (*n* = 3). (**e**) Representative histograms (left) and mean fluorescence intensity (MFI) (right) of lysosomal mass detected by flow cytometry (*n* = 4). (**f**) Epifluorescence microscopy for lysosomal mass (orange staining) and nucleus (blue staining). Scale bar, 20 µM. Results are expressed as fold-change relative to WT fibroblasts. The dotted line represents a WT fibroblast. * *p* < 0.05, ** *p* < 0.005.

The phenotype recovery was evaluated by co-culturing MPS IVA fibroblasts with CRISPR/nCas9-edited CD34+ cells using a transwell-mediated approach (Figure 3a). While untreated MPS IVA fibroblasts exhibited no detectable GALNS enzyme activity (Figure 3b,c), co-culture of MPS IVA fibroblasts with mock-treated CD34+ cells increased the extracellular GALNS enzyme activity (26% WT levels). Conversely, co-culture of MPS IVA fibroblast with CRISPR/nCas9-edited CD34+ cells led to a significant extracellular GALNS enzyme activity, reaching levels comparable to WT fibroblasts (Figure 3b). Similarly, intracellular GALNS activity in MPS IVA fibroblasts co-cultured with CRISPR/nCas9-edited CD34+ cells reached up to 50% of WT levels (Figure 3c). In contrast, co-culture with mock-treated CD34+ cells resulted in only an 8% increase of WT levels in intracellular GALNS enzyme activity (Figure 3c). These results strongly suggest that CRISPR/nCas9-edited CD34+ cells overexpress bioactive GALNS enzyme, which is uptaken by MPS IVA fibroblasts.

Next, we evaluated the impact of these findings on classical pathological biomarkers of MPS IVA [6,51]. While untreated MPS IVA fibroblasts showed an increase in the mono-sulfated KS levels (fold-change: 1.3) compared to WT fibroblasts, MPS IVA fibroblasts co-cultured with CRISPR/nCas9-edited CD34+ cells led to normalization of mono-sulfated KS levels to WT (Figure 3d). Similarly, when evaluating the lysosomal mass via flow cytometry, we found that untreated MPS IVA fibroblasts showed up to a 1.7-fold increase in the lysosomal mass, compared to WT fibroblasts. Upon co-culture with CRISPR/nCas9-edited CD34+ cells, the lysosomal mass in MPS IVA fibroblasts was normalized to WT levels (Figure 3e). Similar findings were noticed in epifluorescence microscopy assays (Figure 3f). These findings strongly suggest that CRISPR/nCas9-edited CD34+ cells can positively impact the lysosomal mass in MPS IVA fibroblasts through cross-correction.

### 2.3. Reduction of Oxidative Stress in MPS IVA Fibroblasts

Alterations in cellular homeostasis and oxidative stress have been identified as critical factors in the pathogeneses of various MPS [51,52,53]. Previously, we reported an improved oxidative profile, particularly in mitochondrial-related oxidative stress (mt-ROS), in MPS IVA [37,38] and GM2 gangliosidoses [54] fibroblasts upon CRISPR/nCas9 treatment. To assess the impact of our CRISPR/nCas9-edited CD34+ cells on the oxidative profile of MPS IVA fibroblasts, we quantified the global- and mt-ROS via flow cytometry after one month. Menadione (vitamin K3), known to induce cell death through ROS-dependent mechanisms, was included as a positive control in these experiments [55].

**Figure 4:**
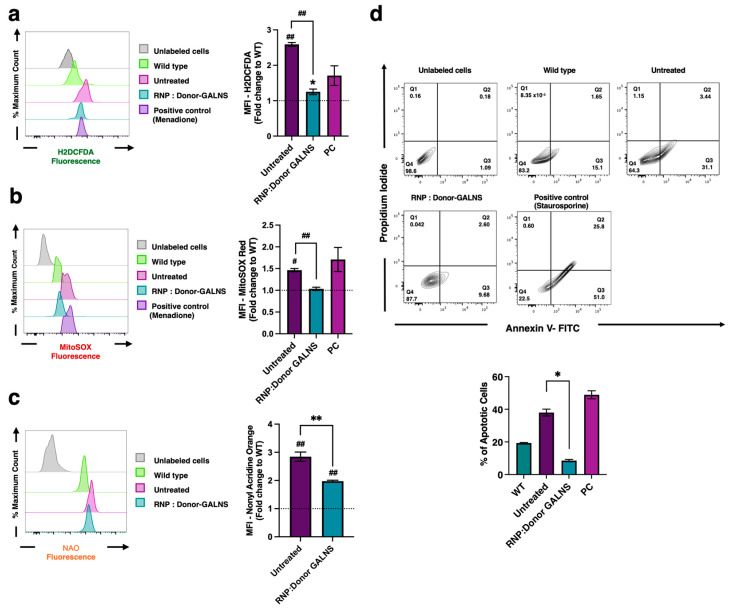
Pro-oxidant profile assessment in MPS IVA fibroblasts co-cultured with CRISPR/nCas9-edited CD34+ cells. (**a**) Detection of reactive oxygen species (ROS). Representative histogram (left) and mean fluorescence intensity (MFI) of H2DCFDA fluorescence probe by flow cytometry (right) (*n* = 6). (**b**) Detection of mitochondrial-related oxidative stress (mtROS). Representative histogram (left) and MFI (right) of MitoSOX red fluorescence by flow cytometry (*n* = 6). (**c**) Detection of mitochondrial mass. Representative histogram (left) and MFI (right) of Nonyl Acridine Orange (NAO) by flow cytometry (*n* = 6). (**d**) Apoptosis assays. Gating strategy (top) and mean percentages of apoptotic cells (bottom) (*n* = 6). Results are expressed as fold-change relative to WT fibroblasts. The dotted line represents WT fibroblasts. PC: Positive control. * *p* < 0.05, ** *p* < 0.005, ^#^ *p* < 0.001, ^##^ *p* < 0.0001.

Untreated MPS IVA fibroblasts were characterized by a significant increase in the global ROS (fold-change: 2.6) compared to WT fibroblasts (Figure 4a). Although co-culture of CRISPR/nCas9-edited CD34+ cells and MPS IVA fibroblasts resulted in a significant 48% reduction in global ROS levels in MPS IVA fibroblasts, they remained significantly higher than those observed in WT fibroblasts (fold-change: 1.3, *p* = 0.0264). Regarding mt-ROS, we observed that untreated MPS IVA fibroblasts showed a 1.4-fold increase compared to WT fibroblasts (Figure 4b). Conversely to global-ROS levels, co-culture of MPS IVA fibroblasts and CRISPR/nCas9-edited CD34+ cells resulted in mt-ROS normalization (Figure 4b).

Growing evidence supports increased mitochondrial mass in some LSDs [36,37,38,54,56,57,58]. To assess the mitochondrial mass in MPS IVA fibroblasts and the impact of our CRISPR/nCas9-based GT approach, we monitored the mitochondrial mass using Nonyl Acridine Orange (NAO) via flow cytometry. Untreated MPS IVA fibroblasts exhibited a ~3-fold increase in mitochondrial mass compared to WT fibroblasts (Figure 4c). Upon co-culture with CRISPR/nCas9-edited CD34+ cells, we observed a reduction in the mitochondrial mass of 28% in co-cultured MPS IVA fibroblasts, compared to untreated MPS IVA fibroblasts (Figure 4c), suggesting amelioration in the mitochondrial mass of MPS IVA fibroblasts.

An increased mitochondrial-related apoptosis ratio was recently reported in MPS IVA fibroblasts [59]. We further tested the apoptosis levels using a flow cytometry approach to assess the effect of co-culturing MPS IVA fibroblasts with CRISPR/nCas9-edited CD34+ cells. Staurosporine induces cell death via intrinsic apoptotic pathways; it was included as a positive control for these experiments [60]. While untreated MPS IVA fibroblasts exhibited increased spontaneous apoptosis (37%), WT fibroblasts showed a basal apoptosis ratio of approximately 19% (Figure 4d). After one month of co-culture, we observed a reduction in apoptosis in MPS IVA fibroblasts to as low as 9% (Figure 4d), suggesting that GALNS enzyme supply via CRISPR/nCas9-edited CD34+ cells ameliorates the pro-apoptotic profile in MPS IVA fibroblasts.

### 2.4. Decreased Levels of Pro-Inflammatory Markers in MPS IVA Fibroblasts

**Figure 5:**
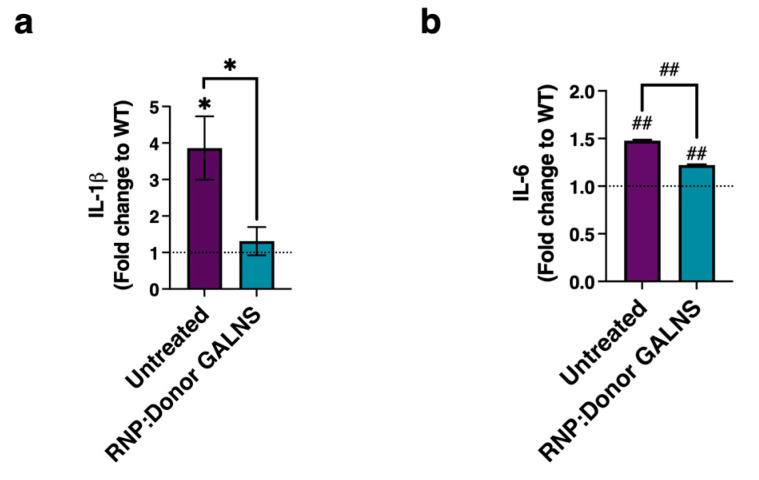
Pro-inflammatory profile evaluation in MPS IVA fibroblasts co-cultured with CRISPR/nCas9-edited CD34+ cells. (**a**) Levels of Interleukin-1 beta (IL-1β) (*n* = 7). (**b**) Levels of Interleukin-6 beta (IL-6) (*n* = 8). Results are expressed as fold-change to WT fibroblasts. The dotted line represents WT fibroblasts. * *p* < 0.05, ## *p* < 0.0001.

Pro-inflammatory factors such as IL-1β and IL-6 are significantly elevated in MPS IVA and other MPS patients [61,62,63,64]. To assess the effect of CRISPR/nCas9-edited CD34+ cells on the pro-inflammatory profile of co-cultured MPS IVA fibroblasts, we performed ELISA to quantify IL-1β and IL-6 (Figure 5a,b). As expected, MPS IVA fibroblasts were characterized by a significant increase in IL-1β (fold-change: 3.9, *p* = 0.0124 ) and IL-6 (fold-change: 1.5, *p* < 0.0001), compared to WT fibroblasts. Upon co-culture, we found that MPS IVA fibroblasts showed decreases of ~67% and ~17% in IL-1β (Figure 5a) and IL-6 levels (Figure 5b), respectively. Collectively, our findings further support the determination that our CRISPR/nCas9 system mediates cross-correction in MPS IVA fibroblasts, ameliorating pro-inflammatory events in MPS IVA fibroblasts.

## 3. Discussion

Previously, we reported a CRISPR/nCas9-based GT strategy for inserting an expression cassette carrying the GALNS cDNA into the AAVS1 locus in human MPS IVA fibroblasts using non-viral vectors [37,38]. Although in vitro assessment showed successful HR of the GALNS cDNA expression cassette, leading to the long-term biochemical recovery of the MPS IVA fibroblast phenotype [37], in vivo assessment, using the ROSA26 locus as a safe harbor, only partially rescued bone lesions in MPS IVA mice [36], highlighting the need for a novel strategy. In this regard, previous studies conducted by Gomez-Ospina et al., using the CRISPR/Cas9 system to edit HSPCs at the CCR5 locus, demonstrated that ex vivo CRISPR/Cas9-based GT could improve bone lesions in MPS I mice upon transplantation [39]. CRISPR/Cas9-edited HSCs can differentiate into diverse blood and immune cell lineages, offering a promising therapeutic approach for systemic pathologies [65]. Recently, ex vivo LV-based GT was tested in MPS IVA mice, demonstrating partial improvement in bone structure [35]. Nevertheless, random integration into the host genome remains a significant concern that needs to be deeply studied [66,67,68].

In this study, we aimed to validate our previously reported CRISPR/nCas9 system in vitro, inserting a GALNS expression cassette at the AAVS1 locus in CD34+ cells. Under our experimental conditions, we observed long-term supraphysiological GALNS levels in CD34+ cells (Figure 1c,d) without affecting stemness (Figure 2). Although the extracellular GALNS levels detected in the CD34+ cells in our study (3-fold change, with respect to unedited cells) were lower than the IDUA levels (25-fold change, with respect to unedited cells) observed by Gomez-Ospina et al., when using PGK as promoter driving gene expression, lysosomal mass was similarly rescued in both approaches upon co-culture with human MPS fibroblasts (Figure 3e,f). While extracellular GALNS enzyme activity was evaluated in bulk CD34+ cells in our study, IDUA activity was quantified in sorted CD34+ cells by Gomez-Ospina et al., potentially explaining the differences between the two studies. Additionally, unlike the IDUA enzyme, which mediates its catabolic activity as a monomer [69,70], GALNS requires homodimerization to catabolize substrates effectively [71], further explaining the differences noticed. Finally, the safe harbors used in our study (AAVS1) and by Gomez-Ospina et al. (CCR5) could also play critical roles in knock-in approaches. Early reports by van Rensburg et al. (2013) showed that chromatin conformation is essential to genome editing efficiency [72]. In this context, van Rensburg et al. reported that while the CCR5 locus and its surrounding regions are characterized by closed chromatin, which hinders Cas9-mediated DNA cutting, the AAVS1 locus remains in open chromatin in stem cells [72], making it a more suitable target for genome editing than is CCR5. GALNS enzyme activity quantification in sorted CD34+ cells will elucidate this premise.

CRISPR/nCas9-edited CD34+ stemness was evaluated, as concerns have been raised about the potential compromise of HSCs’ properties after transfection with CRISPR/Cas9 genome strategies [66]. Indeed, functional assessments of CRISPR/Cas9-edited HSCs are essential [73,74,75]. The CRISPR/nCas9-edited CD34+ cells maintained their stemness, consistent with previous findings in other CRISPR/Cas9-based genome editing studies [39,66,76,77]. Importantly, these results confirm that the CRISPR/nCas9 strategy did not negatively impact the self-renewal or differentiation capabilities of CD34+ cells. Preserving colony-forming potential and multilineage differentiation suggests that CRISPR/nCas9-edited CD34+ cells retain their stemness, which is crucial for robust engraftment [39,46]. More significantly, the preservation of stemness in CRISPR/nCas9-edited CD34+ cells is particularly promising, as edited HSCs could provide sustained therapeutic effects post-transplantation by continuously supplying GALNS-expressing blood cells throughout the body [76,78].

Ex vivo GT for LSDs is based on the ability to release therapeutic levels of the missing lysosomal enzymes to be uptaken by surrounding cells, supplying the deficient enzyme [79]. To evaluate the effect of the GALNS enzyme released by CRISPR/nCas9-edited CD34+ cells, we co-cultured these cells with human MPS IVA fibroblasts for one month and assessed GALNS enzyme activity, along with classical LSD biomarkers. Co-culturing CRISPR/nCas9-edited CD34+ cells with MPS IVA fibroblasts resulted in a recovery of the intracellular GALNS enzyme activity of up to 50% WT levels (Figure 3c). These results agreed with our previous findings on MPS IVA fibroblasts using non-viral vectors [38].

Consistent with the recovery of GALNS enzyme activity, we observed efficient cross-correction in MPS IVA fibroblasts upon co-culture with CRISPR/nCas9-edited CD34+ cells. Specifically, we noted a reduction in pathological markers such as KS accumulation and lysosomal mass, both of which were normalized to WT levels (Figure 3d). The reduction in mono-sulfated KS achieved in this study was comparable to previous in vitro studies, in which the CRISPR/nCas9 system led to normalization of total GAGs in human MPS IVA fibroblasts [37,38]. A consistent decrease in lysosomal mass was observed in MPS IVA fibroblasts co-cultured with CRISPR/nCas9-edited CD34+ cells (Figure 3e,f). These findings agreed with previous findings when using CRISPR/nCas9- [37,38] and LV-based GT [56].

Increased pro-oxidant levels and mitochondrial mass are critical biomarkers for evaluating mitochondrial dysfunction in several MPS [36,37,38,51,56,57,58,80,81]. Since mitochondria are the primary source of ROS, excessive ROS production is associated with mitochondrial dysfunction [82,83]. Notably, co-culturing MPS IVA fibroblasts with CRISPR/nCas9-edited CD34+ cells resulted in an improvement in global ROS levels while normalizing mtROS levels to WT levels (Figure 4a,b). Furthermore, our results demonstrated a reduction in mtROS levels compared to those reported in fibroblasts treated with a non-viral CRISPR/nCas9-strategy GT [37,38].

LSDs are characterized by lysosomal dysfunction; therefore, it is reasonable to hypothesize that impaired autophagy/mitophagy could increase mitochondrial mass [58,84]. In line with this assumption, we observed a significant increase in mitochondrial mass in untreated MPS IVA fibroblasts (Figure 4c). When MPS IVA fibroblasts were co-cultured with CRISPR/nCas9-edited CD34+ cells, a significant decrease in the mitochondrial mass was detected, suggesting that lysosomal mass recovery may also influence lysosome-related organelles such as mitochondria. Likewise, mitochondrial dysfunction strongly triggers intrinsic apoptosis [85,86]. Under our experimental conditions, we found that untreated MPS IVA fibroblasts exhibited a significant pro-apoptotic profile, which can be reverted upon co-culture with CRISPR/nCas9-edited CD34+ cells (Figure 4d); this further supports the determination that supplying the GALNS enzyme through ex vivo CRISPR/Cas9-based GT could be a promising approach for preserving cell survival. Notably, the increase in apoptosis levels was recently reported by Brokowska et al. (2023) [59], supporting our findings for untreated MPS IVA fibroblasts.

Pro-inflammatory events have been described in MPS IVA patients [87,88], and they seem not to be ameliorated upon ERT treatment [63]. To assess the pro-inflammatory profile in MPS IVA fibroblasts and evaluate its modulation upon co-culture with CRISPR/nCas9-edited CD34+ cells, we quantified the levels of IL-1β and IL-6 through ELISA (Figure 5). Both interleukins were significantly elevated in untreated MPS IVA fibroblasts; however, co-culture with CRISPR/nCas9-edited CD34+ cells led to significant recovery as to these parameters, suggesting a positive effect on the pro-inflammatory profile. The impact of our CRISPR/nCas9-based GT in HSCs on the pro-inflammatory profile, mitochondrial mass recovery, and pro-apoptotic profile still needs to be further addressed in MPS IVA/CD34 + cells and MPS IVA animal models.

This study provides substantial evidence for the therapeutic potential of CRISPR/nCas9-edited HSCs in treating MPS IVA. By editing CD34+ cells to introduce a functional GALNS gene at the AAVS1 locus, we achieved sustained GALNS enzyme expression, efficient cross-correction in co-cultured MPS IVA fibroblasts, and a recovery of disease-related biomarkers, including GAG accumulation, oxidative stress, apoptosis ratio, and pro-inflammatory profile. The CRISPR/Cas9-edited HSCs maintained their self-renewal and differentiation capabilities, which are crucial for ensuring long-term therapeutic benefits following transplantation.

Overall, the results from this study highlight the promising therapeutic potential of CRISPR/nCas9-edited HSCs as an effective ex vivo gene therapy for MPS IVA. The ability of these CRISPR/nCas9-edited HSCs to differentiate into various blood cell lineages and permanently to secrete the GALNS enzyme throughout the body supposes therapeutical effects in several affected organs via cross-correction. Further pre-clinical in vivo studies in MPS IVA mouse models will lead to a complete evaluation of the safety, efficacy, and clinical applicability of this promising therapeutic strategy before its translation into clinical practice.

## 4. Materials and Methods

### 4.1. Cell Culture and Expansion

Human bone marrow CD34+ stem cells were purchased from StemCell Technologies (Vancouver, BC, Canada) and cultured in StemSpan™ SFEM II medium supplemented with 1X StemSpan™ CD34+ Expansion Supplement [77] and 1 µM small molecule UM729 [89]. CD34+ were maintained at 37 °C in 5% CO_2_ for 3 days to allow sufficient cell expansion before gene editing. Human WT and MPS IVA fibroblasts were obtained from Nemours Children’s Health Biobank (IBC #1237) and cultured in Dulbecco’s Modified Eagle Medium (DMEM, Gibco^®^, Waltham, MA, USA) supplemented with 20% fetal bovine serum (FBS) (Gibco^®^) and 1% penicillin-streptomycin (Gibco^®^) at 37 °C and 5% CO_2_ for 2 days before co-culture with CD34+. Co-culture was performed in Corning Transwell^®^ (Corning, NY, USA) clear inserts of 0.4 µm pore size polyethylene Terephthalate (PET) membrane and maintained for 30 days at 37 °C, 5% CO_2_.

### 4.2. Donor Template

The donor template was designed according to our previous protocols, with slight modifications [37,54]. Briefly, two arms homologous to the AAVS1 locus comprising 500 bp each flanked an expression cassette containing the human phosphoglycerate kinase (hPGK) promoter, a Kozak sequence, GALNS cDNA, and the bovine growth hormone (bGH) poly(A) signal sequence (AAV6-donor). In some experiments, an additional donor carrying the enhanced green fluorescent protein (EGFP) sequence upstream of GALNS cDNA was designed (AAV6-donor-EGFP). The P2A sequence was added to mediate ribosome skipping between EGFP and GALNS. Donor templates were delivered to cells using adeno-associated virus type 6 (AAV6). Vectors were synthesized by VectorBuilder Inc. (Chicago, IL, USA).

### 4.3. CRISPR/nCas9 Editing Protocol

CD34+ cells were electroporated with a ribonucleoprotein (RNP) complex composed of Cas9-D10A nickase protein (nCas9) (Sigma-Aldrich, Rockville, MD, USA) and PUREedit™ modified sgRNAs targeting the AAVS1 locus (synthesized by Sigma-Aldrich). The two sgRNA target sequences were previously reported [37,38,54]. Briefly, after 3 days post-thawing, 1 × 10^6^ CD34+ cells were resuspended in the RNP complex at a molar ratio of 1:2.5 (nCas9: sgRNA) at room temperature and subsequently electroporated using the 4D-Nucleofector X (Lonza, Walkersville, MD, USA) with the P3 Primary Cell Kit. Following electroporation, the AAV6 donor template was delivered at a multiplicity of infections (MOI) of 15,000. Additionally, CD34+ cells electroporated with either the RNP complex or donor template (mock) were included as experimental controls.

### 4.4. Donor Template Integration at the AAVS1 Locus

Donor template integration at the AAVS1 locus was evaluated by the EGFP expression in CD34+ cells electroporated with or without the RNP complex, followed by transduction of AAV6-donor-EGFP. EGFP expression was assessed by flow cytometry after 30 days using a NovoCyte 3000 cytometer (Agilent Technologies, Santa Clara, CA, USA). Data were analyzed by FlowJo^TM^ 10.10.0 Software (Becton Dickinson, Franklin Lakes, NJ, USA).

### 4.5. Stemness Evaluation

#### 4.5.1. CD Markes

The expression profile of primitive hematopoietic progenitor markers was analyzed in CRISPR/nCas9-edited CD34+ after 14 days post-edition. Cells were stained with anti-human CD34+ APC antibody (1:50, Cat. 130-113-176, Miltenyi Biotec, Bergisch Gladbach, Germany), anti-human CD38+ PE antibody (1:50, Cat. 130-113-431, REAfinity™, Miltenyi Biotec), and anti-human CD45+ FITC antibody (1:50, Cat. 130-110-631, Miltenyi Biotec). A total of 10,000 viable single cells were analyzed using a NovoCyte 3000 cytometer (Agilent Technologies). Gating strategy for the CD34+, CD38+, and CD45+ analysis is shown in Supplementary Figure S3. Isotype Control Antibody, mouse IgG2a, APC (1:50, Cat. 130-113-269, Miltenyi Biotec), REA Control Antibody (S), human IgG1, PE (1:50, Cat. 130-113-438, Miltenyi Biotec), and Isotype Control Antibody, mouse IgG2a, FITC (1:50, Cat. 130-113-279, Miltenyi Biotec) were used as isotype controls.

#### 4.5.2. Colony-Forming Unit (CFU)

CFU experiments were performed to evaluate differentiation potential in CRISPR/nCas-edited CD34+ cells. At 15 days post-editing, CRISPR/nCas9-edited CD34+ cells were seeded in MethoCult^TM^ media on SmartDish^TM^ culture plate dishes (StemCell Technologies, Vancouver, BC, Canada) at a concentration of 5000 cells/mL and up to 14 days of incubation followed. The resulting colonies were classified as colony-forming unit-erythroid (CFU-E); colony-forming unit-granulocyte, erythrocyte, macrophage, megakaryocyte (CFU-GEMM); Burst-forming unit-erythroid (BUF-E); and colony-forming unit-granulocyte, macrophage (CFU-GM) [39,77]. Colony classification was conducted using visual inspection aided by inverted microscopy (AXIO Observer Z1 microscope, Zeiss, Jena, Germany). Additionally, a flow cytometry-based analysis was included to evaluate the differentiation potential of CRISPR/nCas9 edited CD34+ cells, using the StemMACS^TM^ HSC-CFU Assay Kit (Miltenyi Biotec), following the manufacturer’s instructions. The total number of colonies and colony type were determined based on the percentages of positive signals for CD15, CD14, and CD235a differentiation markers in viable single cells. The colony type was classified based on whether cells exhibited a single positive signal for one marker or positive signals for two or more markers, analyzed in a NovoCyte 3000 cytometer (Agilent Technologies). The data obtained were analyzed by FlowJo^TM^ 10.10.0 Software (Becton Dickinson).

#### 4.5.3. Proliferation and Cell Cycle Analysis

The proliferation index and cell cycle of CRISPR/nCas9-edited CD34+ cells were analyzed after 14 days post-editing using 7-Aminoactinomycin D (7-AAD) (Invitrogen^TM^, Waltham, MA, USA) and Hoechst 33342 (Invitrogen^TM^, Waltham, MA, USA), respectively, following a previously reported protocol, with minor modifications [90]. Briefly, the cells were collected and washed by centrifugation at 90× *g* for 5 min with 1X Hank’s Balanced Salt Solution (1X HBSS, Gibco^®^) supplemented with 2% FBS (Gibco^®^). The cells were then fixed in 200 µL of 70% ice-cold ethanol for 1 h at 4 °C, followed by a wash with 1X HBSS. Subsequently, the cells were permeabilized using PTF buffer (Phosphate buffered saline (PBS), 0.25% Triton X-100, and 1% FBS). Finally, the cells were stained in 200 µL of 12.5 µg of 7-AAD- or 10 µg/mL Hoechst-containing 1X HBSS and incubated for 30 min at 4 °C in the dark. At least 50,000 events were collected using excitation/emission (exc/emi): 650 nm (7AAD) and a 453 nm long-pass filter on the NovoCyte 3000 cytometer (Agilent Technologies). The resulting data were analyzed using FlowJo^TM^ 10.10.0 Software (Becton Dickinson).

### 4.6. GALNS Activity Assays

Specific and volumetric GALNS enzyme activity in MPS IVA fibroblasts and CRISPR/nCas9-edited CD34+ cells were determined as previously described [36,91]. GALNS activity was measured using 4-MU-Gal-6S (22 nM; Toronto Chemicals Research, Vaughan, ON, Canada). The reaction was incubated at 37 °C for 17 h, followed by incubation with 2 µL of β-galactosidase (10 mg/mL; Sigma-Aldrich) for one hour. The enzyme reaction was stopped by 968 µL of glycine-carbonate buffer (pH 9.8). The plate was read using exc/emi: 365/450 nm in a FLUOstart Omega microplate reader (BMG LabTech, Ortenberg, Germany). Enzyme activity (U) was defined as the amount of enzyme required to hydrolyze 1 nmol of substrate per hour, expressed either per milliliter (U mL^−1^) or per milligram of protein (U mg^−1^), based on a 4-methylumbelliferone standard curve (Sigma-Aldrich). Protein concentration was quantified using a BCA Protein Assay Kit (Thermo Fisher Scientific, Waltham, MA, USA). Data are presented as fold-change relative to untreated.

### 4.7. Lysosomal Accumulation

#### 4.7.1. Lysosomal Mass Evaluation

After 30 days of culturing, the lysosomal mass in MPS IVA fibroblasts co-cultured with CRISPR/nCas9-CD34+ cells was determined by staining fibroblasts with LysoTracker Deep Red (Invitrogen^TM^), as previously described [36,37,38]. Briefly, fibroblasts were incubated with 50 nM LysoTracker in DMEM supplemented with 20% FBS for 1 h. Then, the cells were collected by trypsinization and washed twice with 1X PBS; this was followed by gentle resuspension in a solution containing 1:1000 Propidium Iodine (PI; Invitrogen^TM^) in 1X HBSS plus 2% FBS. Finally, 50,000 events of viable single fibroblasts were analyzed using a NovoCyte 3000 cytometer (Agilent Technologies) using exc/emi: 647/668 nm. The data were analyzed using FlowJo^TM^ 10.10.0 Software (Becton Dickinson). The lysosomal mass was further investigated by epifluorescence microscopy, as previously reported, with minor modifications [36,37]. MPS IVA fibroblasts were seeded on coverslips pre-coated with 50 µg Poly-D-Lysine (Gibco^®^) and co-cultured with CRISPR/nCas9-edited CD34+ cells for 30 days. The cells were then incubated with 75 nM LysoTracker Deep Red for one hour, washed twice with 1X PBS, and fixed in 4% paraformaldehyde in PBS (Invitrogen^TM^) for 15 min at room temperature. The coverslips were finally transferred to slides containing Prolong Glass Antifade Mountant with NucBlue Stain (Invitrogen^TM^). Images were captured using inverted fluorescence microscopy (AXIO Observer Z1 microscope) and analyzed using ImageJ 1.54p software [92].

#### 4.7.2. Analysis of Mono-Sulfated Keratan Sulfate

Mono-sulfated KS levels in MPS IVA fibroblasts co-cultured with CRISPR/nCas9-edited CD34+ cells were determined by liquid chromatography–tandem mass spectrometry (LC-MS/MS) via enzymatic digestion of KS polymers, as previously described [36,93]. Protein extracts from fibroblasts, obtained using Pierce^®^ Ripa buffer (Thermo Fisher Scientific), were processed with 96-well Omega 10K molecular weight cutoff filter plates (Pall Corporation, Port Washington, NY, USA) positioned on a 96-well receiving plate. A reaction mixture containing 50 nM Tris-HCl (pH 7.0), 5 µg/mL chondrosine as the internal standard (IS), 1 mU heparitinase, and 1 mU Keratanase II was added to each well. The plate was incubated overnight at 37 °C. After incubation, the samples were centrifuged at 2500× *g* for 15 min. Disaccharides were isolated using a Hypercarb column (2.0 mm inner diameter, 50 mm length, 5 µM particle size; Thermo Fisher Scientific) at 60 °C. A binary gradient elution of 5 nM ammonium acetate (PH 11.0) transitioning to 100% acetonitrile was used. The mass spectrometer (Agilent Technologies) was operated in negative ion mode with electrospray ionization at a flow rate of 0.7 mL/min and a runtime of 5 min. Disaccharides were detected using m/z ratios, with precursor and product ions for IS (354.3 → 193.1) and mono-sulfated KS (462 → 97).

### 4.8. Oxidative Profile Assessment

#### 4.8.1. Reactive Oxygen Species (ROS)

Global oxidative stress was assessed by incubating MPS IVA fibroblasts, co-cultured for one month with CRISPR/nCas9-edited CD34+ cells, in 5 µM H2DCFDA (2′,7′-dichlorodihydrofluorescein diacetate, Invitrogen^TM^) diluted in DMEM supplemented with 20% FBS. The incubation was performed at 37 °C for 30 min. Afterward, the cells were harvested by trypsinization, washed twice with 1X PBS, and resuspended in 1X HBSS. Subsequently, the cells were stained with 0.1 µg/mL of PI and incubated at room temperature for 30 min. Finally, 10,000 viable single cells were analyzed using a NovoCyte 3000 cytometer (Agilent Technologies, Santa Clara, CA, USA) with an exc/emi: 585/504 nm wavelength. Fibroblasts incubated with 100 µM menadione were used as a positive control [55]. The data were analyzed using FlowJo^TM^ 10.10.0 Software (Becton Dickinson)

#### 4.8.2. Mitochondrial-Derived Reactive Oxygen Species (mtROS)

The mitochondrial-dependent oxidative stress in MPS IVA fibroblasts co-cultured with CRISPR/nCas9- and mock-treated CD34+ cells was measured one month after incubation, using MitoSOX^TM^ mitochondrial superoxide indicator (Invitrogen^TM^), as described before [37,54]. Briefly, fibroblasts were stained with 5 µM MitoSOX^TM^ diluted in DMEM supplemented with 20% FBS for 15 min at 37 °C. The cells were then collected by trypsinization, washed twice with 1X PBS, and resuspended in 1X HBSS. Fibroblasts treated with 100 µM of Menadione (Sigma-Aldrich) were included as positive controls [55]. Finally, 10,000 events of viable single fibroblasts were analyzed using a NovoCyte 3000 cytometer (Agilent Technologies) with an exc/emi: 510/580 nm wavelength. The data were analyzed using FlowJo^TM^ 10.10.0 Software (Becton Dickinson).

### 4.9. Mitochondrial Mass Detection

Mitochondrial mass was evaluated in MPS IVA fibroblasts co-cultured with CRISPR/nCas9-edited CD34+ cells using Nonyl Acridine Orange (NAO, Invitrogen^TM^), exc/emi: 488/530 nm. Briefly, MPS IVA fibroblasts were incubated with 200 nM NAO for one hour at 37 °C. After two washes with 1X HBSS, the cells were used for flow cytometry experiments using a NovoCyte 3000 cytometer (Agilent Technologies). Cell viability was evaluated by PI staining. The data were analyzed using FlowJo^TM^ 10.10.0 Software (Becton Dickinson).

### 4.10. Apoptosis Ratio

MPS IVA fibroblasts co-cultured with CRISPR/nCas9-edited CD34+ cells were used to quantify the apoptosis ratio using Dead Cell Apoptosis Kits with Annexin V assay (Molecular Probes, Eugene, OR, USA) to quantify phosphatidyl serine (PS) externalization. PS was detected using an Alexa Fluor 488-conjugated Annexin V antibody (exc/emi: 488/530 nm). Fibroblasts incubated with 1 μM staurosporine for 6 h were used as a positive control of apoptosis [59,60]. The data were analyzed using FlowJo^TM^ 10.10.0 Software (Becton Dickinson)

### 4.11. Pro-Inflammatory Profile Evaluation

Enzyme-linked immunosorbent assay (ELISA) assays were conducted to evaluate the levels of IL-1β (BMS224-2, Invitrogen^TM^) and IL-6 (BMS213HS, Invitrogen^TM^) in MPS IVA fibroblasts co-cultured with CRISPR/nCas9-edited CD34+ cells. Briefly, an aliquot of media was collected from MPS IVA fibroblasts for one month and stored at −20 °C. Then, samples were pooled, clarified by centrifugation, and used to determine cytokine levels per the manufacturer’s instructions. The plates were read using a FLUOstart Omega microplate reader (BMG LabTech, Ortenberg, Germany). The absorbance values from the MPS IVA fibroblasts were plotted against a standard curve for each cytokine.

## 5. Statistical Analysis

Statistical tests were processed and analyzed using GraphPad Prism software version 10.4.1 for macOS (GraphPad Software, LLC, San Diego, CA, USA). The data are reported as mean ± standard error (SE). Shapiro–Wilk and Levene’s tests were used to evaluate the normal distribution and homoscedasticity. According to normal distribution findings, mean comparisons between groups were made with Student’s *t*-test, the Mann–Whitney U test, or one-way analysis of variance (ANOVA).

## Data Availability

All available data are published.

## Supplementary Materials

The following supporting information can be downloaded at: https://www.mdpi.com/article/10.3390/ijms26094334/s1.

## Abbreviations

The following abbreviations are used in this manuscript:

MPS	Mucopolysaccharidosis
LSD	Lysosomal storage disease
GAGs	Glycosaminoglycans
KS	Keratan sulfate
C6S	Chondroitin 6-sulfate
GALNS	N-acetylgalactosamine-6-sulfatase
GT	Gene therapy
ERT	Enzyme replacement therapy
HSC	Hematopoietic stem cell
HSCT	Hematopoietic stem cell transplantation
GVHD	Graft versus host disease
PC	Pharmacological chaperones
SDT	Substrate degradation therapy
WT	Wild type
EGFP	Enhanced green fluorescent protein
