# Supplementary material for: CRISPR/nCas9-Edited CD34+ Cells Rescue Mucopolysaccharidosis IVA Fibroblasts Phenotype
Source: Int J Mol Sci. 2025 May 2;26(9):4334. doi: 10.3390/ijms26094334 (PMC12072265; doi:10.3390/ijms26094334)
Supplement: Supplementary file 1 [file ijms-26-04334-s001.zip › ijms-3566989-supplementary.pdf]

Supplemental Information

CRISPR/nCas9-Edited CD34+ Cells Rescue Mucopolysaccharidosis IVA Fibroblasts Phenotype

Angélica María Herreno-Pachón [1,2,†], Andrés Felipe Leal [1,3,†], Shaukat Khan [1], Carlos Javier Alméciga-Díaz [3] and Shunji Tomatsu [1,4,5,*]

[1] Nemours Children's Health, Wilmington, DE 19803, USA; angelicamaria.herrenopachon1@nemours.org (A.M.H.-P.); andres.lealbohorquez@nemours.org (A.F.L.); shaukat.khan@nemours.org (S.K.)
[2] Faculty of Arts and Sciences, University of Delaware, Newark, DE 19716, USA
[3] Institute for the Study of Inborn Errors of Metabolism, Faculty of Science, Pontificia Universidad Javeriana, Bogotá 110231, DC, Colombia; cjalmeciga@javeriana.edu.co
[4] Department of Pediatrics, Graduate School of Medicine, Gifu University, Gifu 501-1193, Japan
[5] Department of Pediatrics, Thomas Jefferson University, Philadelphia, PA 19107, USA
* Correspondence: shunji.tomatsu@nemours.com; Tel.: +1-302-298-7336; Fax: +1-302-651-6888
† These authors contributed equally to this work.

Supplementarily Materials and Methods

On-target efficiency of CRISPR/nCas9 RNP complex

On-target cutting at the AAVS1 *locus* was conducted using the T7 endonuclease assay in CRISPR/nCas-edited HEK-293 cells. Briefly, genomic DNA was isolated aided by Monarch™ Genomic DNA purification kit (New England Biolabs, Ipswich, MA) according to the manufacturer's instructions. As previously reported [1], a PCR was conducted to amplify the AAVS1 *locus* with specific primers (the primers sequences can be consulted in [1]. Following the manufacturer's instructions, the amplicon was digested with the EnGen® Mutation Detection Kit (New England Biolabs). The digestion product was visualized in 2% agarose gel stained with 0.5 µg of ethidium bromide. The resulting images were analyzed with GelAnalizer 19.1, as previously described [2], to determine the INDEL frequency. Next, HEK-293 cells were transduced with AAV6-Donor-EGFP and maintained for up to 30 days, and expression of EGFP was measured through flow cytometry in a NovoCyte 3000 cytometer (Agilent Technologies, Santa Clara, CA). Data were analyzed by FlowJo 10.10.0 Software (Becton Dickinson, Franklin Lakes, NJ).

References

1. Leal, A.F. and C.J. Almeciga-Diaz, Efficient CRISPR/Cas9 nickase-mediated genome editing in an in vitro model of mucopolysaccharidosis IVA. Gene Ther, 2023. 30(1-2): p. 107-114.
2. Chiang, T.W., et al., CRISPR-Cas9(D10A) nickase-based genotypic and phenotypic screening to enhance genome editing. Sci Rep, 2016. 6: p. 24356.

Supplementarily Results

a

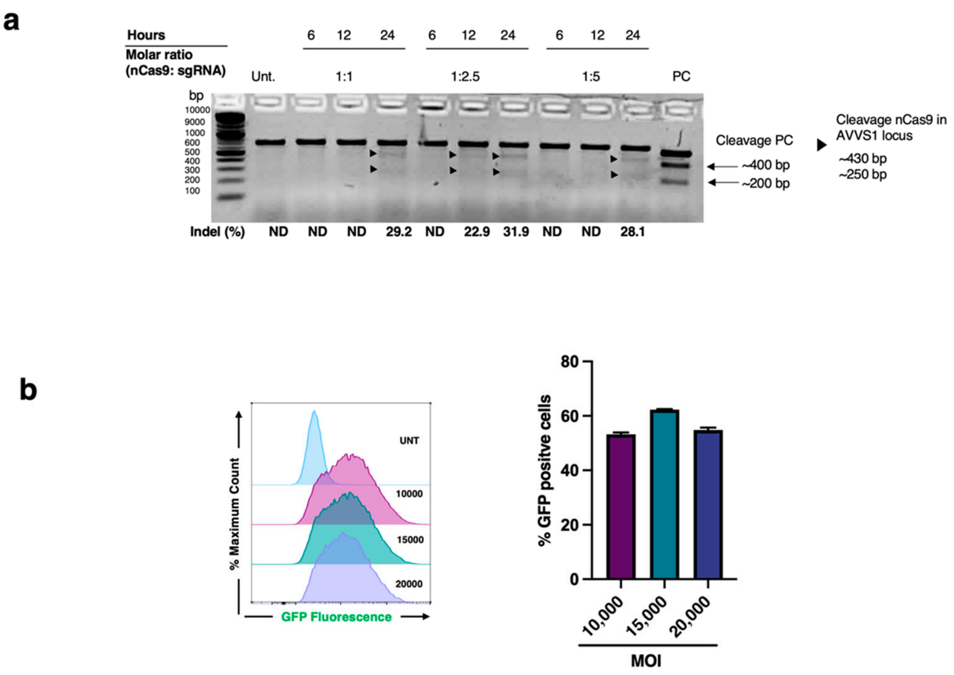

Supplementary Figure S1. On-target efficiency of CRISPR/nCas9 RNP complex. (a): Agarose gel showing the on-target efficiency of sgRNAs targeting the AAVS1 locus in HEK-293 cells detected by T7-base endonuclease assay. PC (positive control). **(b):** Transduction efficiency of AAV6-Donor-EGFP at different MOIs. Representative histogram of GFP-positive HEK-293 cells (left) and mean of GFP-positive HEK-293 cells.

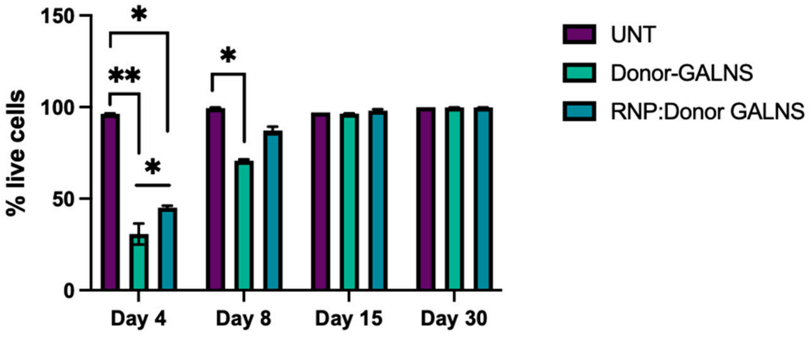

Supplementary Figure S2. Cell viability. Percentage of live cells post-editing in CD34+ cells transfected with RNP-based CRISPR/nCas9 and AAV6-donor alone. Cell viability was evaluated by PI in flow cytometry. *$p < 0.05$, **$p < 0.005$

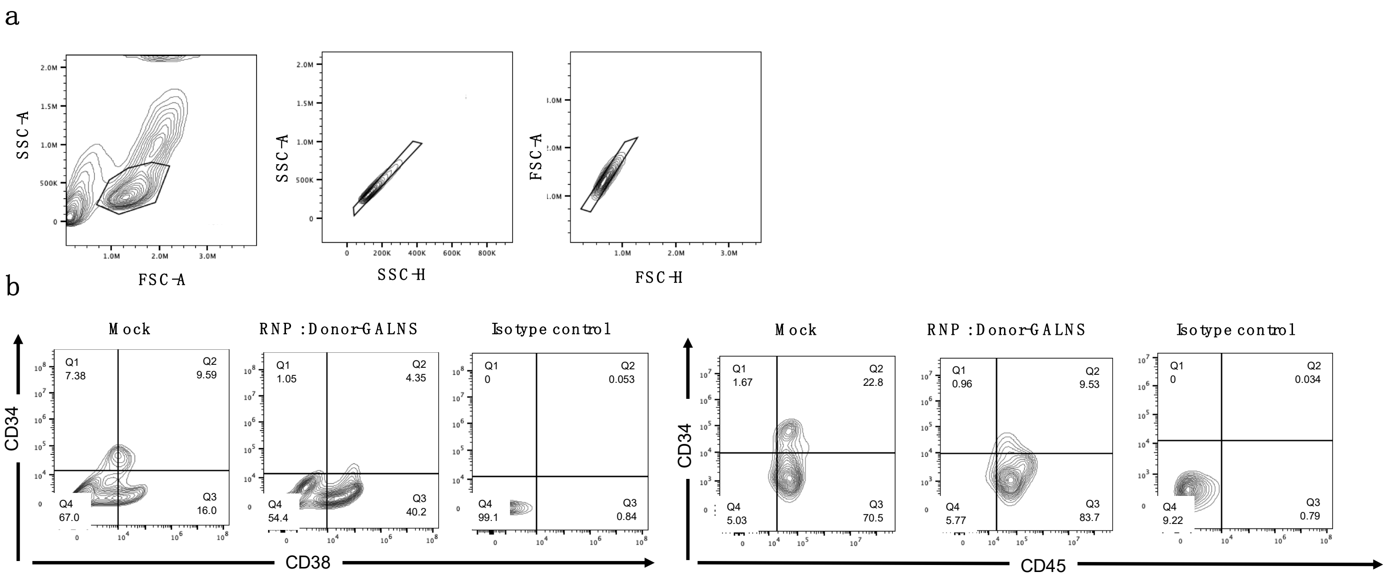

Supplementary Figure S3. Gating strategy of CD34+ cells. Representative gating strategy for analyzing CD34+, CD38+, and CD45+ markers in CD34+ cells transfected without or with RNP-based CRISPR/nCas9 system.
